# Immunopeptidome profiling in pulmonary fibrosis: linking antigenicity to cytotoxic immunity and therapeutic targeting

**DOI:** 10.1038/s41392-026-02871-6

**Published:** 2026-07-30

**Authors:** Theodoros Karampitsakos, Brenda M. Juan-Guardela, Jose D. Herazo-Maya

**Affiliations:** 1 First Department of Pulmonary, Critical Care and Sleep Medicine, Hospital for Thoracic Diseases ‘’SOTIRIA”, National and Kapodistrian University of Athens, Athens, Greece; 2 Department of Internal Medicine, Division of Pulmonary, Critical Care and Sleep Medicine, Ubben Center for Pulmonary Fibrosis Research, Morsani College of Medicine, University of South Florida, Tampa, FL USA

**Keywords:** Antigen processing and presentation, Respiratory tract diseases

In a paper recently published in Nature Immunology, Bai et al. provided robust evidence that immunopeptidome profiling could serve as a platform for discovering translatable antifibrotic immunotherapies. The authors showed that overcoming T cell dysfunction through the induction of antigen-specific cytotoxic T lymphocytes represents a potent antifibrotic mechanism.^1^

This study further supports the role of immunity in lung fibrosis. Currently, pathogenesis of lung fibrosis is widely considered to involve a complex interplay of environmental exposure, genetics, epigenetics, metabolic reprogramming, dynamic remodeling of extracellular matrix, and immune aberrations.^2^ However, historically, the contribution of the immune system to fibrogenesis was underappreciated, in part due to the adverse outcomes observed with corticosteroid therapy in idiopathic pulmonary fibrosis (IPF).^3^ Accumulating evidence now supports a central role for immune dysregulation in disease pathogenesis.^4,5^ Among the earliest studies supporting this paradigm shift was the identification of a 52-gene peripheral blood signature capable of predicting mortality across six independent IPF cohorts.^5^ Subsequent cellular deconvolution analyses demonstrated that monocytes are the primary source of the upregulated genes, prompting increased focus on the role of monocytes and macrophages in lung fibrogenesis. More recently, advances in single-cell RNA sequencing have enabled the identification of discrete monocyte subpopulations that drive disease progression.^4^ In particular, CD14⁺CD163⁻HLA-DR^low^ monocytes were found to be strongly associated with IPF severity, progression, and mortality across peripheral blood, bronchoalveolar lavage, and lung tissue compartments. These cells exhibit a profibrotic, proangiogenic, and chemotactic transcriptional program, supporting their differentiation into pathogenic lung macrophages. Notably, CD14⁺CD163⁻HLA-DR^low^ monocytes also display an immunosuppressive phenotype. Patients enriched for the corresponding 230-gene signature demonstrate reduced expression of T-cell co-stimulatory pathways, suggesting a state of T-cell exhaustion or “immunoparalysis” that may contribute to fibrosis progression. Importantly, transcriptomic profiling of this population has also revealed candidate therapeutic targets through Connectivity Map (CMap) analysis, highlighting potential avenues for precision-based intervention.^4^

These observations are further complemented by the elegant study of Bai et al, which demonstrated that overcoming T cell dysfunction represents a potent antifibrotic mechanism (Fig. 1). The authors systematically mapped the MHC class I immunopeptidome in IPF and presented data that reframe fibrosis as an antigenically tractable disease. Analysis of both human and murine samples revealed a diverse, disease-specific immunopeptidome enriched for peptides derived from pathogenic myofibroblasts and profibrotic macrophages. Importantly, these findings were extended into functional relevance. Selected candidate epitopes were tested in vivo, and vaccination with peptides, including those derived from macrophage-associated factors (MAFs), significantly attenuated bleomycin-induced pulmonary fibrosis. Notably, MAF-derived peptides elicited human cytotoxic T lymphocyte responses capable of lysing IPF-derived myofibroblasts and M2-like macrophages.^1^ These findings are highly consequential. In the context of accumulating evidence that T-cell exhaustion is a central feature of lung fibrosis, the ability to restore or elicit effective cytotoxic T-cell responses suggests a potentially powerful antifibrotic mechanism. The demonstration of both in vivo efficacy and relevance to human immune responses further underscores the strong translational potential of this approach.

**Fig. 1:**
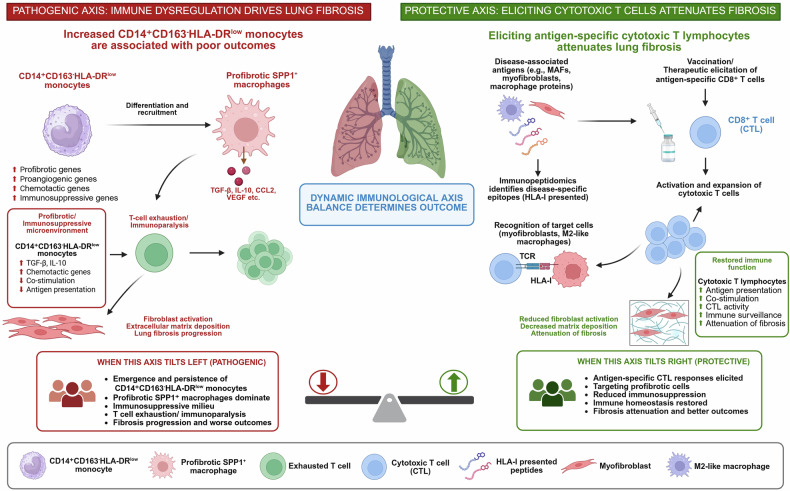
Schematic representation of immune aberrations in lung fibrosis. CD14⁺CD163⁻HLA-DR^low^ monocytes exhibit a profibrotic, proangiogenic, and chemotactic transcriptional program, supporting their differentiation into pathogenic lung macrophages. Notably, these cells also display an immunosuppressive phenotype. The aforementioned promote fibrogenesis. On the other hand, overcoming T cell dysfunction represents a potent antifibrotic mechanism. *Abbreviations: CCL2: chemokine (C-C motif) ligand 2, IL-10: Interleukin 10, MAFs: macrophage-associated factors, SPP1: Secreted Phosphoprotein 1, TCR: T cell receptor, TGF-b: Transforming growth factor beta, VEGF: Vascular endothelial growth factor. Note: The figure has been created in* https://BioRender.com

Beyond these immediate implications, the study also opens important avenues for future research and therapeutic development. High-resolution immunopeptidomics, coupled with computational clustering and motif analysis, revealed distinct binding signatures and source-protein enrichments in fibrotic compared to non-fibrotic tissues. This level of analytical resolution enables not only the identification but also the prioritization of biologically meaningful epitopes—addressing a major bottleneck in immunotherapy development. A particularly promising future direction is the integration of immunopeptidomics with Connectivity Map (CMap) analysis. While immunopeptidomics defines antigen presentation at the peptide level, CMap captures perturbation-induced gene expression signatures.^1,4^ Future computational approaches capable of validating findings at the peptide and gene-expression levels may increase the likelihood of identifying clinically efficacious compounds, ultimately facilitating precision-targeted therapeutic strategies in patients with immune-driven disease phenotypes.^2^

Another promising avenue for future investigation is the integration of immunopeptidomic data with high-resolution single-cell and spatial transcriptomic approaches to precisely define the cellular sources and spatial context of antigen presentation in fibrosis. The study of Bai et al.^1^ provides compelling evidence that the expanded HLA class I immunopeptidome in human IPF is largely derived from proteins associated with tissue remodeling by fibroblasts and macrophages. Multicolor immunofluorescence further demonstrated that CD8⁺ T cells localize in close proximity to α-SMA⁺ myofibroblasts within fibrotic lesions in the lungs of MAF_116–124_ vaccinated bleomycin-instilled mice. While these findings are highly informative, it remains to be addressed which precise cellular subsets are involved and the stages of differentiation at which they participate during antigen presentation and immune engagement. Single-cell RNA sequencing can be leveraged to map the expression of peptide source proteins alongside antigen-processing and presentation machinery across diverse stromal and immune cell populations, thereby enabling the identification of fibroblast and macrophage subsets that most actively contribute to the immunopeptidome. Incorporating trajectory inference and RNA velocity analyses would enable reconstruction of fibrotic progression and help define the temporal window during which antigenic signatures emerge. In parallel, cell–cell communication frameworks such as CellChat may uncover signaling networks that promote or sustain antigen presentation, linking immune-derived cues to stromal activation states. Critically, spatial transcriptomics could further resolve the co-localization of antigen-presenting cells within fibrotic niches. The integration of these modalities would transform static peptide catalogs into dynamic, spatially resolved maps of antigen presentation, ultimately delineating the contribution of each cellular mediator to the feed-forward T cell immunosuppressive loop that characterizes lung fibrosis. For example, such an approach could delineate whether transforming growth factor-beta, along with other mediators such as interleukin-10, drives the emergence and persistence of CD14^+^CD163^−^HLA-DR^low^ monocytes and profibrotic macrophages, culminating in T cell exhaustion, or whether an alternative temporal sequence underlies these events. Conversely, this mechanistic framework not only delineates the pathogenic immune circuitry but also opens avenues for therapeutic reprogramming. Targeted immunotherapies such as CAR-T therapy or drugs aiming to decrease profibrotic monocytes and macrophages are greatly anticipated.

Taken together, the study of Bai et al.^1^ highlights that pulmonary fibrosis is a condition in which endogenous antigenicity exists but is insufficiently harnessed, likely due to deficient antigenic presentation by circulating monocytes and tissue macrophages. These findings provide a mechanistic framework for immunopeptidome-driven therapeutic strategies aimed at restoring effective cytotoxic immune surveillance. More broadly, they reinforce the concept that targeting immune dysregulation is a critical component of a comprehensive approach to the treatment of lung fibrosis.

## References

[1] Bai, Z. et al. Immunopeptidome profiling in pulmonary fibrosis provides a platform for identifying therapeutic targets. *Nat. Immunol.* **27**, 923–936 (2026). 42010059 10.1038/s41590-026-02501-x PMC13132728

[2] Karampitsakos, T., Tourki, B. & Herazo-Maya, J. D. The dawn of precision medicine in fibrotic interstitial lung disease. *Chest* **167**, 120–132 (2025). 10.1016/j.chest.2024.10.042 PMC12001815 39521375

[3] The Idiopathic Pulmonary Fibrosis Clinical Research Network. Prednisone, azathioprine, and N-acetylcysteine for pulmonary fibrosis. *N. Engl. J. Med*. **366**, 1968–1977 (2012). 10.1056/NEJMoa1113354 PMC3422642 22607134

[4] Karampitsakos, T. et al. The transcriptome of CD14(+)CD163(-)HLA-DR(low) monocytes predicts mortality in Idiopathic Pulmonary Fibrosis. *Eur. Respir. J.* **67**, 2500804 (2026). 41360505 10.1183/13993003.00804-2025 PMC13150356

[5] Herazo-Maya, J. D. et al. Validation of a 52-gene risk profile for outcome prediction in patients with idiopathic pulmonary fibrosis: an international, multicentre, cohort study. *Lancet Respir. Med.* **5**, 857–868 (2017). 28942086 10.1016/S2213-2600(17)30349-1 PMC5677538

